# Complete genome sequence of a porcine circovirus type 2 strain, PCV2/CN/GD/2018/10, obtained in Guangdong, China, in 2018

**DOI:** 10.1128/mra.01003-23

**Published:** 2024-01-30

**Authors:** Lihua Cao, Wenke Lv, Xingyao Feng, Lisha Chen, Lulu Yang, Jinyue Guo

**Affiliations:** 1 Department of Preventive Veterinary Medicine, College of Life Science and Engineering, Foshan University, Foshan, Guangdong, China; DOE Joint Genome Institute, Berkeley, California, USA

**Keywords:** PCV2, genome analysis

## Abstract

Porcine circovirus type 2 (PCV2) poses significant issue for the global swine industry. We conducted a comprehensive analysis of the complete genome sequence of a Chinese PCV2 strain belonging to genotype PCV2a, which was designated as PCV2/CN/GD/2018/10. Our findings provide insights into the prevalence of PCV2 in China.

## ANNOUNCEMENT

Porcine circovirus is a globally distributed pathogen that poses significant harm to the swine industry. Currently, four circoviruses have been identified, namely, PCV1, porcine circovirus type 2 (PCV2), PCV3, and PCV4 (1–4). Among them, PCV2 is known to be the causative agent of various porcine circovirus associated diseases, including post-weaning multisystem wasting syndrome, swine dermatitis and nephrotic syndrome, porcine respiratory disease complex, myocarditis/vasculitis and exudative dermatitis, intestinal disease, and reproductive failure (5–9).

PCV2 is a non-enveloped, single-stranded circular DNA virus belonging to the Circoviridae family and the *Circovirus* genus family (10). The PCV2 genome contains two major open reading frames: ORF1 and ORF2. ORF1 codes for the replicase protein and is fundamental for viral replication; ORF2 codes for the capsid protein involved in viral attachment and represents the main target of the host immune response (11). PCV2 has been classified into eight genotypes: PCV2a–PCV2h (12). Additionally, a novel genotype, PCV2i, was recently identified in the United States (13). However, only PCV2a, PCV2b, and PCV2d exhibit high incidence globally and appear variably over time (14).

In March 2018, lung tissue sample was obtained from a pig farm in Qingyuan city, Guangdong province, China, which tested PCV2 positive based on the conventional PCR, as the established protocol in the Preventive Veterinary Medicine Laboratory of Foshan University (15). A PCV2, named PCV2/CN/GD/2018/10, was identified. To determine the complete genome sequence of PCV2/CN/GD/2018/10, lung tissue supernatant DNA was extracted using the AxyPrep Virus DNA/RNA kit (AXYGEN Biotechnology, Hangzhou, China). The whole-genome sequence was amplified via conventional PCR using specific primers (Table 1). Subsequently, the PCR products were purified and cloned into the PMD 18-T vector (TaKaRa, Dalian, China). Positive clones were sent to an outsourced service (Sangon Biotech, Shanghai, China) for sequencing using Sanger’s method. The complete genome sequence of the PCV2/CN/GD/2018/10 strain was assembled using the SeqMan software in DNAStar Lasergene v.10.2014 (DNAStar, Inc., Madison, WI).

**TABLE 1:** Primer sequences used for amplification of the complete PCV2 genome

Primer name	Primer sequence	Product size(bp）
PCV2-F	5′-GGGCTGGCTGAACTTTTGAAAGTGAGC-3′	1,768
PCV2-R	5′-CCAGCCCGCGGAAATTTCTGACAAACG-3′	

**Fig 1:**
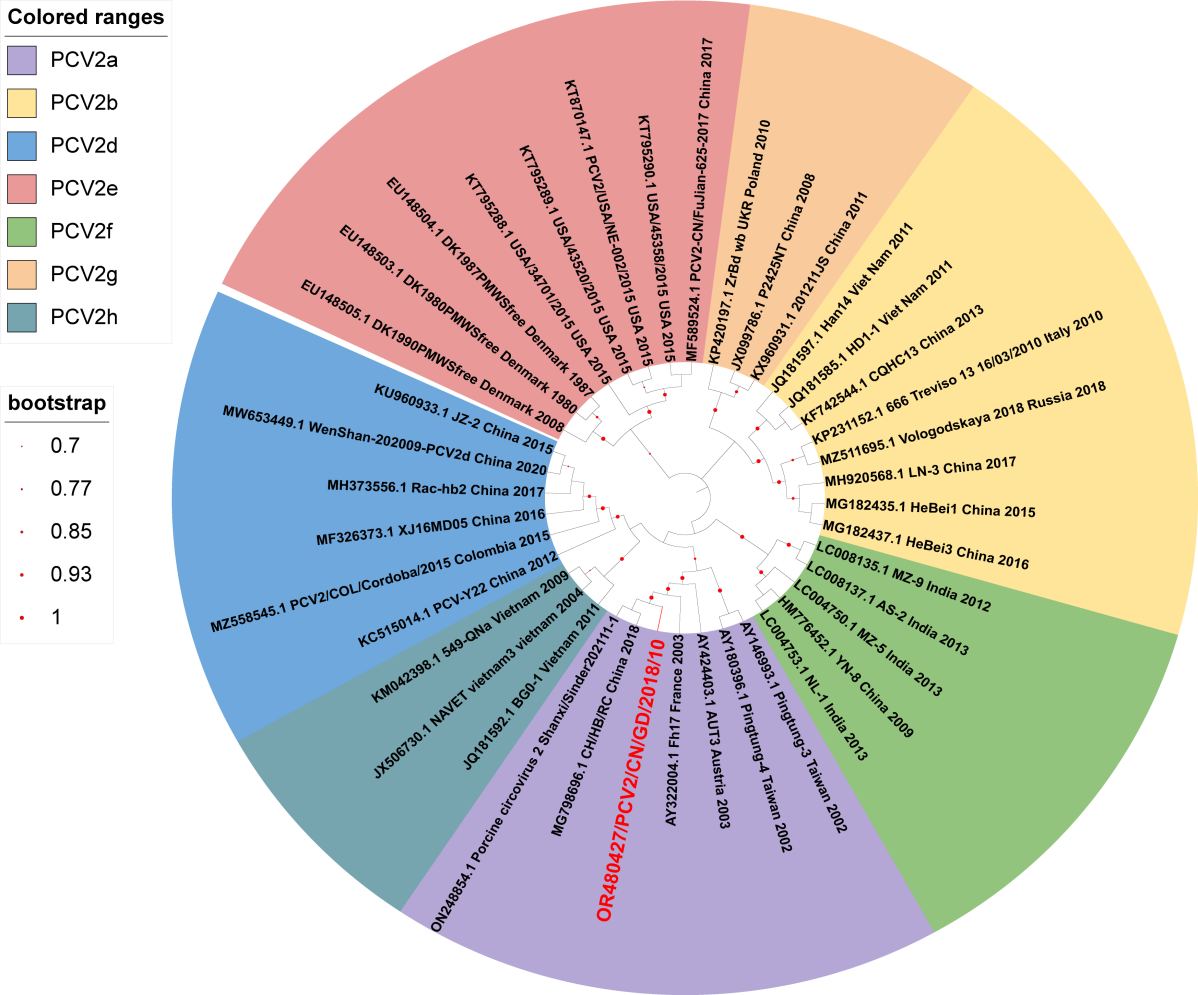
Phylogenetic tree of the complete nucleotide genome sequence of PCV2. The phylogenetic trees were constructed using the maximum likelihood method and general time reversible model of MEGA X based on 1,000 bootstrap replicates. The location of our strain sequence is marked with red font in the purple area of the figure.

The complete nucleotide genome sequence of the PCV2/CN/GD/2018/10 strain was subjected to phylogenetic analysis using MEGA X software, along with 39 reference strains from the GenBank database. The sequence analysis indicated that the genome of the obtained strain had a length of 1,768 nucleotides with an approximate GC content of 48.36%. ORF1 comprised 945 nucleotides, encoding a protein of 314 amino acids, while ORF2 consisted of 702 nucleotides, encoding a protein with 234 amino acids. Further similarity analysis of these sequences at the nucleotide sequence level, using BioAider v.1.527 software, showed that this strain exhibited the highest similarity (99.83%) with PCV2/CN/Shanxi/Sinder202111-1 (GenBank accession number ON248854.1) . It displayed 97.04%–100.0% identity in the ORF1 gene and 79.5%–99.57% identity in the ORF2 gene with other reference strains at the nucleotide sequence level. Phylogenetic analysis of the whole genome revealed that the genome belongs to the PCV2a genotype (Fig. 1). These findings will aid in the epidemiological analysis of PCV2a and contribute to the development of new vaccines.

## Data Availability

The complete genome sequences of PCV2/CN/GD/2018/10 are available at GenBank under the accession number OR480428.1. The raw sequence of Sanger reads has been deposited in the Sequence Read Archive under Bio Project accession number PRJNA1047954.
